# Constructing a Watts-Strogatz network from a small-world network with symmetric degree distribution

**DOI:** 10.1371/journal.pone.0179120

**Published:** 2017-06-12

**Authors:** Mozart B. C. Menezes, Seokjin Kim, Rongbing Huang

**Affiliations:** 1 Operations Management Department, Kedge Business School - Bordeaux, 680 cours de la Libération, 33405 Talence, France; 2 Department of Information Systems and Operations Management, Sawyer Business School, Suffolk University, 120 Tremont Street, Boston, MA 02108, United States of America; 3 School of Administrative Studies, York University, 4700 Keele Street, Toronto, ON, Canada M3J 1P3; Universidad Rey Juan Carlos, SPAIN

## Abstract

Though the small-world phenomenon is widespread in many real networks, it is still challenging to replicate a large network at the full scale for further study on its structure and dynamics when sufficient data are not readily available. We propose a method to construct a Watts-Strogatz network using a sample from a small-world network with symmetric degree distribution. Our method yields an estimated degree distribution which fits closely with that of a Watts-Strogatz network and leads into accurate estimates of network metrics such as clustering coefficient and degree of separation. We observe that the accuracy of our method increases as network size increases.

## Introduction

Since the term “small world” was coined first by the Milgram’s pioneering experiment [1], Watts and Strogatz [2] have proposed the most compelling analytical framework demonstrating the small-world phenomenon prevalent in a range of social, information, technological, and biological networks. A small world consists of many local clusters, but all members are connected with short distance via a few more connected members. These conditions for a small world to emerge are minimal and many real networks have shown small-world properties [3–5]. However, it is not straight-forward to quantify “small-world-ness.” A quantitative model measures the equivalence between a network and a unique Watts-Strogatz (WS) model [6]. For a real network with high equivalence, the corresponding WS model can be generated to explore the structure and dynamics.

Even though such equivalence is confirmed, it is not viable to study a large real network when sufficient data on a population are not available. For example, modern social networks are very large in sizes and it takes significant resources and time to collect the data of a network and find key parameters. Web-based experiments accommodating a large number of participants are more difficult to control in some respects than are those conducted in physical laboratories [7]. When such real experiments are impractical, an artificially structured network can be studied instead [8]. However, a large real network with strong small-world properties cannot be replicated into the corresponding WS model, unless its parameters are estimated from a sample.

A WS model [2] is characterized by *n* = number of nodes, *K* = number of neighbors a node has to its right side in the regular lattice before rewiring, and *p* = “rewiring” probability with which the right end of an arc incident to a node is rewired uniformly randomly to another node. The size of population under study is represented by *n* which is known a priori or can be estimated [9, 10] in many cases. The sample mean of node degrees is an estimator for *K* since the total number of arcs remain invariant after rewiring. Among the three parameters, it is the most challenging to estimate *p*. Motivated by this immediate need, we formulate a method to estimate *p* leading into an estimated degree distribution which fits closely with that of the corresponding WS model. These three parameters (*n*, *K*, *p*) indeed suffice to characterize a WS network. We observe that, from many generated WS networks under the same values of (*n*, *K*, *p*), variations in network metrics such as clustering coefficient (CC) and degree of separation (DS) (defined as characteristic path length in [2]) are very small.

A direct question from this motivation, then, is how many arcs are incident to node *i* ∈ *N* after rewiring, where *N* is the set of nodes in a network. We start from deriving the degree distribution of a network represented by the probability *P*(*δ*_*i*_ = *m*) that node *i* has a degree of *m*, where *P* is a probability mass function and *δ*_*i*_ = the degree of node *i* (or the number of arcs incidents to node *i*) after rewiring. A previous derivation of *P*(*δ*_*i*_ = *m*) in [11] is based on the assumption that *δ*_*i*_ ≥ *K* after rewiring, which might not be the case for some WS networks we have generated. Nonetheless, this assumption allows a simpler formulation as a result. We thus propose a new formulation of *P*(*δ*_*i*_ = *m*) which is closer to the exact value in a WS network.

## Results and discussion

In the regular lattice of a WS network before rewiring, a node *i* ∈ *N* has degree 2*K* with *K* arcs incident to its right neighbors and *K* to its left ones. Let *N*_*i*_ be the set of nodes connected to node *i* before or during rewiring (whereas *δ*_*i*_ is the degree of node *i* after rewiring). Then, before rewiring, |*N*_*i*_| = 2*K*. Note that *N*_*i*_ does not include node *i*. Node *i* loses one degree after a sequence of events below takes place to node *j* ∈ *N*_*i*_ with the assumption of |*N*_*i*_| = 2*K*.

1 Arc {*i*, *j*} is chosen for rewiring with probability *p*.
2 One end of arc {*i*, *j*} (attached to node *i*) is chosen with probability 1/2.
3 The chosen end is rewired, with probability (*n* − 1 − 2*K*)/*n*, to a node which is neither node *j* nor one of the nodes in *N*_*i*_.

Consequently, the probability that the degree of a node decreases by 1 is $\alpha \equiv \frac{1}{2}\;p\;\frac{\left(n-1-2K\right)}{n}$. We admit that |*N*_*i*_| = 2*K* might not be the case during rewiring and our formulation of *P*(*δ*_*i*_ = *m*) is an approximation. The small world property (high local clustering and short paths) emerges for a small rewiring probability *p* ranging from 0.001 to 0.1 in Fig 2 in [2]. For a small *p*, e.g., *p* = 0.01, about 1% of the arcs are rewired. Accordingly, the degree of most nodes would be *N*_*i*_ = 2*K* during rewiring and this assumption is not significantly limiting. As shown in the examples we have generated, our approximation still results in small errors.

On the other hand, node *i* gains one degree after the steps below if arc {*j*, *k*}, among the (*n* − 2)*K* arcs not incident initially to node *i*, is detached and an end of arc {*j*, *k*} is rewired to node *i* chosen randomly. If *i* = *j* or *i* = *k*, arc {*j*, *k*} is attached back and not rewired.

4 Arc {*j*, *k*} is detached for rewiring with probability *p*.
5 Node *i* is chosen with probability 1/*n* and an end of arc {*j*, *k*} is rewired to node *i*.

Thus, the probability that the degree of a node increases by 1 is *β* ≡ *p*/*n*.

We rewrite the number of degrees after rewiring as *δ*_*i*_ = 2*K* − *X*_*i*_ + *Y*_*i*_, where *X*_*i*_ and *Y*_*i*_ are binomial random variables representing the number of degrees lost and the number of degrees gained at node *i*, respectively. Then we have

$$\begin{array}{ccc} P(X_{i}=x) & = & \left(\begin{array}{c} 2K\\ x \end{array}\right)\;\alpha^{x}\;{\left(1-\alpha \right)}^{2K-x},\;\text{for}\;x=0,1,...,2K; \end{array}$$ (1)

$$\begin{array}{ccc} P(Y_{i}=y) & = & \left(\begin{array}{c} (n-2)K\\ y \end{array}\right)\beta^{y}\;{\left(1-\beta \right)}^{(n-2)K-y},\;\text{for}\;y=0,1,...,\left(n-2\right)K. \end{array}$$ (2)

and

$$\begin{array}{ccc} P(\delta_{i}=m) & = & P(2K-X_{i}+Y_{i}=m) \end{array}$$ (3)

$$\begin{array}{ccc} & = & \sum_{x=\max\{2K-m,0\}}^{\min\{n-1-m,2K\}}P\left(Y_{i}=m-2K+x\;|\;X_{i}=x\right)\;P\left(X_{i}=x\right). \end{array}$$ (4)

For *m* ≥ *n* or *m* < 0, *P*(*δ*_*i*_ = *m*) = 0.

In Eq (4), we have bounds as max{2*K* − *m*, 0} ≤ *x* ≤ min{*n* − 1 − *m*, 2*K*}, where *x* is the number of degrees lost. For 0 ≤ *m* ≤ 2*K*, 2*K* − *m* ≤ *x* ≤ 2*K*. Node *i* loses at most 2*K* degrees since |*N*_*i*_| = 2*K* before rewiring, but cannot lose less than 2*K* − *m* degrees. Otherwise, *δ*_*i*_ = *m* is impossible. For 2*K* < *m* ≤ *n* − 1 − 2*K*, 0 ≤ *x* ≤ 2*K*. In this case, node *i* can lose all 2*K* degrees as long as it can gain from the other *n* − 1 − 2*K* nodes and can lose none since 2*K* < *m*. For *n* − 1 − 2*K* < *m* ≤ *n* − 1, 0 ≤ *x* ≤ *n* − 1 − *m*. Since *m* is larger than in the previous case, node *i* loses no more than *n* − 1 − *m* degrees and can lose none. In the conditional probability in Eq (4), *Y*_*i*_ = *m* − 2*K* + *x* is immediate from 2*K* − *x* + *Y*_*i*_ = *m*.

We assume a large *n* (e.g., *n* >>*K*) consistent with a large network which we are mainly interested in. From Eqs (1), (2) and (4), we have

$$\begin{array}{ccc} P(\delta_{i}=m) & = & \sum_{x=\max\{2K-m,0\}}^{\min\{n-1-m,2K\}}\left(\begin{array}{c} 2K\\ x \end{array}\right)\alpha^{x}\;\;{\left(1-\alpha \right)}^{2K-x}\;\times \\ & & \left(\begin{array}{c} (n-2)K\\ m-2K+x \end{array}\right)\beta^{\left(m-2K+x\right)}{\left(1-\beta \right)}^{\left((n-2)K-(m-2K+x)\right)}. \end{array}$$ (5)

The probability mass function of binomial distribution with probability *β* in Eq (5) can be approximated by that of a Poisson distribution with rate *λ* = (*n* − 2)*Kβ* = (*n* − 2)*Kp*/*n* for a large *n* and a small *β*, which are the cases in small-world networks. From the fact that $\alpha =\frac{1}{2}\;p\;\frac{\left(n-1-2K\right)}{n}\to \frac{p}{2}$ and $\lambda =\frac{\left(n-2\right)}{n}Kp\to Kp$ for a large *n*, Eq (5) can be written as

$$\begin{array}{c} P\left(m\right)=P\left(\delta_{i}=m\right)=\sum_{x=\max\{2K-m,0\}}^{\min\{n-1-m,2K\}}\left(\begin{array}{c} 2K\\ x \end{array}\right){\left(\frac{p}{2}\right)}^{x}\;\;{\left(1-\frac{p}{2}\right)}^{2K-x}\times \frac{{\left(Kp\right)}^{m-2K+x}}{(m-2K+x)!}e^{-Kp}. \end{array}$$ (6)

Then the mean estimated from Eq (6) is $\tilde{\mu }=\sum_{m=0}^{n}mP\left(m\right)$ and the standard deviation is $\tilde{\sigma }=\sqrt{\sum_{m=0}^{n}{\left(m-\tilde{\mu }\right)}^{2}P\left(m\right)}$. From now on, we use interchangeably use *P*(*m*) and *P*(*δ*_*i*_ = *m*).

**Fig 1:**
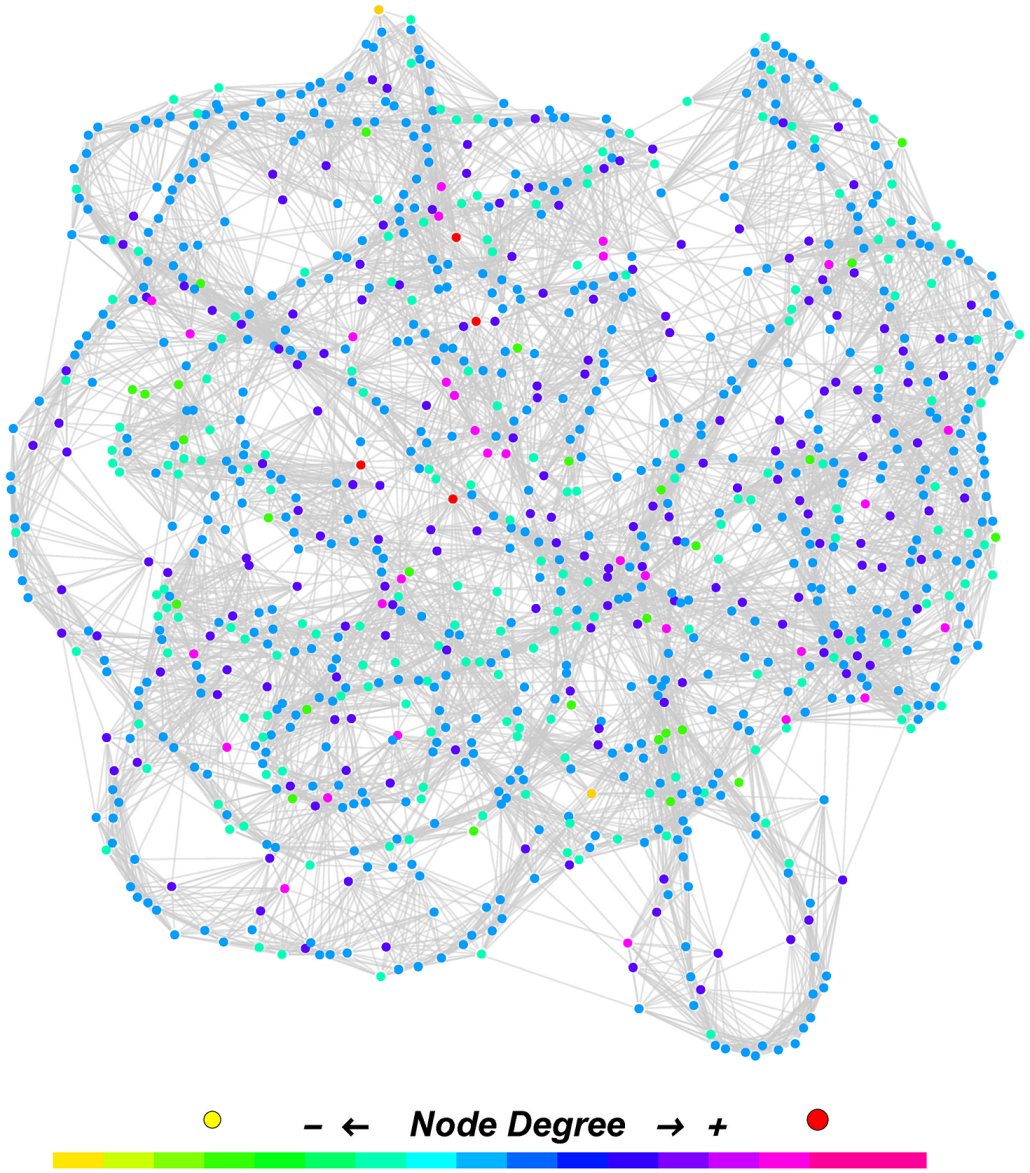
A Watts-Strogatz network. This example with parameters of (*n* = 1,000, *K* = 8, *p* = 0.04), generated in Mathematica 10 by Wolfram Research, has *CC* = 0.6170 and *DS* = 4.1531. Nodes are distinguished by different sizes and colors representing their degrees ranging from from 13 to 19.

**Fig 2:**
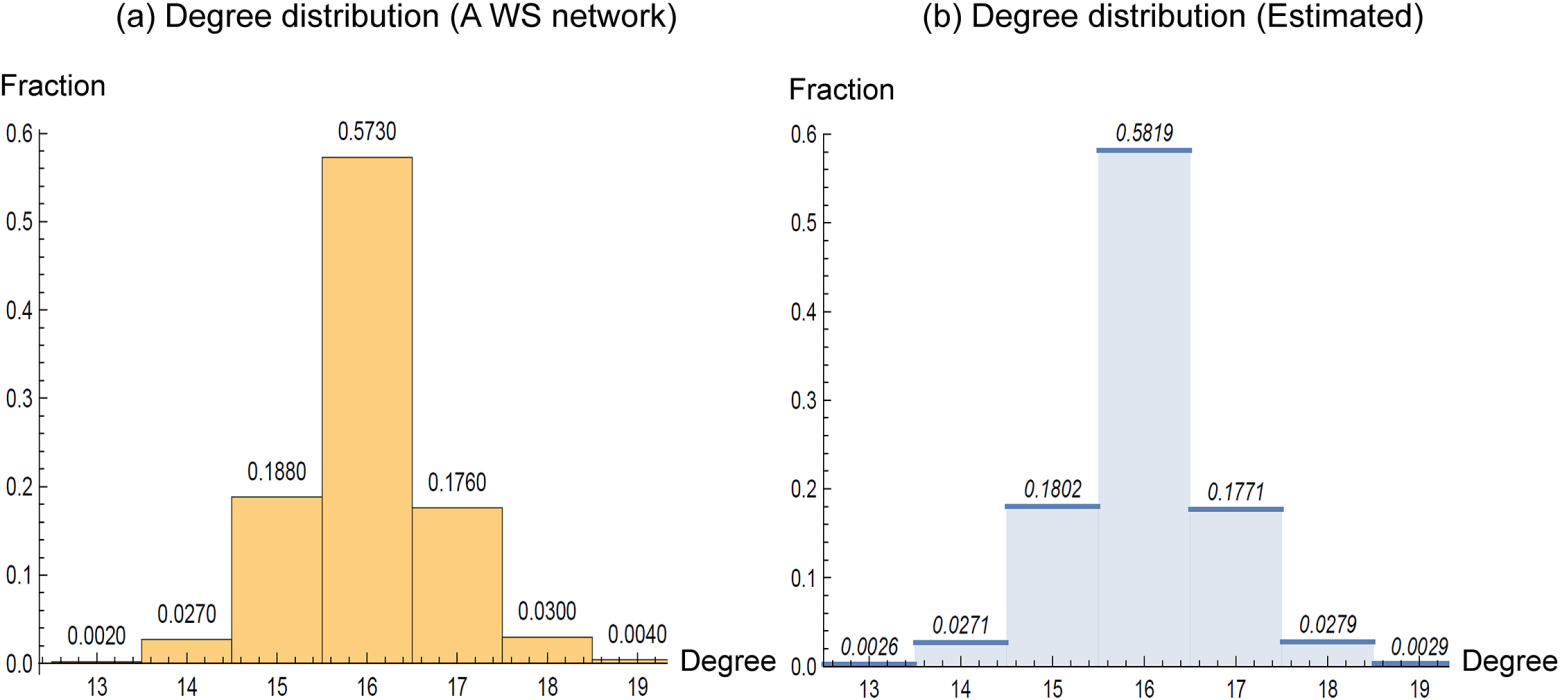
Distributions of degrees in a Watts-Strogatz network and degrees estimated by Eq (6). The WS network was generated with parameters of (*n* = 1,000, *K* = 8, *p* = 0.04).

Fig 1 includes an actual WS network generated with parameters of (*n* = 1,000, *K* = 8, *p* = 0.04). As shown in Fig 2, the degree distribution of the generated WS network is symmetric and closely estimated by Eq (6). Due to the symmetry of degrees in WS networks, our framework is intended for networks with symmetric degree distribution.

The example WS network in Fig 1 is not the only one whose node degrees are close to those estimated by Eq (6). We now demonstrate their statistical fit via 8 tuples of parameters which were set to be *n* = 5,000, 10,000, *K* = 50, 75 and *p* = 0.01, 0.05. For each tuple of (*n*, *K*, *p*), 100 WS networks were generated and their node degrees were recorded for two tests performed. First, a chi-square test was performed for each of 800 WS networks between the actual node degrees and estimated values (*nP*(*δ*_*i*_ = *m*)) given by Eq (6). None of the 800 tests were significant at the given significance level of *α* = 5% and these results corroborate our observation in Fig 2. Second, a *t*-test was performed on the number of nodes *for each degree* *m* for 100 WS networks with respect to each tuple of (*n*, *K*, *p*). The 95% confidence interval is ${\hat{\mu }}_{m}\pm t_{0.025}\left({\hat{\sigma }}_{m}/\sqrt{100}\right)$, where ${\hat{\mu }}_{m}$ is the sample mean from 100 WS networks and ${\hat{\sigma }}_{m}$ is the sample standard deviation. For each tuple and each *m*, the value of *nP*(*δ*_*i*_ = *m*) estimated by Eq (6) lied within the corresponding confidence interval.

**Fig 3:**
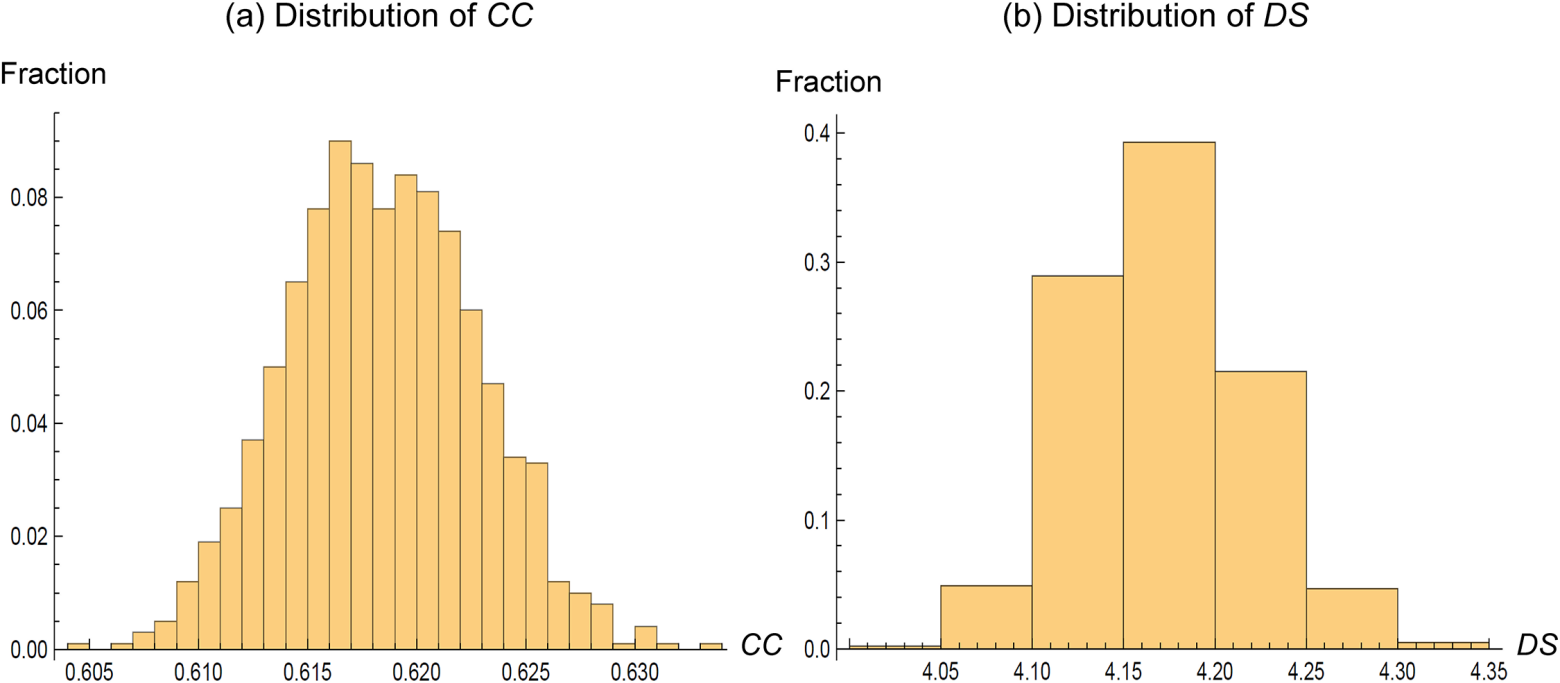
Distributions of *CC* and *DS*. 1,000 WS networks were randomly generated with parameters of (*n* = 1000, *K* = 8, *p* = 0.04).

Fig 3 shows the distributions of *CC* and *DS* from 1,000 WS networks randomly generated with parameters of (*n* = 1,000, *K* = 8, *p* = 0.04) kept the same as in Fig 1. The distributions of *CC* and *DS* are symmetric with small ranges (0.6048 ≤ *CC* ≤ 0.6336 and 4.0278 ≤ *DS* ≤ 4.3333). Thus, given estimates of *n*, *K* and *p* are accurate, the resulting estimates of *CC* and *DS* would also be accurate. It is promising to use our method to estimate *K* and *p* from a sample and then evaluate network metrics such as *CC* and *DS* of the corresponding WS network.

Given that *n* is known or estimated, we propose an algorithm below to find estimates $\hat{K}$ and $\hat{p}$ for their population values of *K* and *p*, respectively. Let *S* = {(*i*, *δ*_*i*_); *i* = 1, …, *s*} be a set of *s* individuals sampled from a WS network, where individual *i* has a degree of *δ*_*i*_. Since the total number of arcs remains the same after rewiring, an estimate for the sample mean is $\hat{K}=\left(\sum_{i=1}^{s}\delta_{i}\right)/\left(2s\right)$ and the sample standard deviation is estimated to be $\hat{\sigma }=\sqrt{{\left(\sum_{i=1}^{s}\delta_{i}-2\hat{K}\right)}^{2}/\left(s-1\right)}$. Then we perform a search for $\hat{p}$ until the standard deviation ($\tilde{\sigma }\left(\hat{p}\right)=\sqrt{\sum_{m=0}^{n}{\left(m-\tilde{\mu }\right)}^{2}P\left(m\right)}$, where $\tilde{\mu }=\sum_{m=0}^{n}mP\left(m\right)$) calculated from Eq (6) gets close enough to $\hat{\sigma }$. Our algorithm is based on the key observation that, as the rewiring probability increases in the WS procedure, the variations of degrees also increase in the resulting network. Thus, given $\tilde{\sigma }\left(\hat{p}\right)$ from Eq (6), we find $\hat{p}$ in a “reverse” manner.

**Algorithm 1**

*Input*: *n*, (*δ*_*i*_, *i* = 1, …, *s*)

Define *ϵ* with a very small value (e.g., 0.00001).

Calculate $\hat{K}=\sum_{i=1}^{s}\delta_{i}/\left(2s\right)$ and $\hat{\sigma }=\sqrt{\sum_{i=1}^{s}{\left(\delta_{i}-2\hat{K}\right)}^{2}/\left(s-1\right)}$.

Let *p*_*l*_ = 0, *p*_*u*_ = 1 and $\hat{p}=\left(p_{l}+p_{u}\right)/2$.

Given $\hat{p}$, use Eq (6) to calculate $\tilde{\mu }=\sum_{m=0}^{n}mP\left(m\right)$ and $\tilde{\sigma }\left(\hat{p}\right)=\sqrt{\sum_{m=0}^{n}{\left(m-\tilde{\mu }\right)}^{2}P\left(m\right)}$.

**while** $\tilde{\sigma }\left(\hat{p}\right)\notin \left[\hat{\sigma }-\epsilon ,\;\hat{\sigma }+\epsilon \right]$ **do**

 If $\tilde{\sigma }\left(\hat{p}\right)>\hat{\sigma }$, let $p_{u}=\hat{p}$. Else, let $p_{l}=\hat{p}$.

 Let $\hat{p}=\left(p_{l}+p_{u}\right)/2$ and use Eq (6) to calculate $\tilde{\mu }$ and $\tilde{\sigma }\left(\hat{p}\right)$.

**end while**

Construct a WS network with parameters of $\left(n,\hat{K},\hat{p}\right)$, and calculate *CC* and *DS*.

$Output:(n,\hat{K},\hat{p}),CC,\;DS$

**Fig 4:**
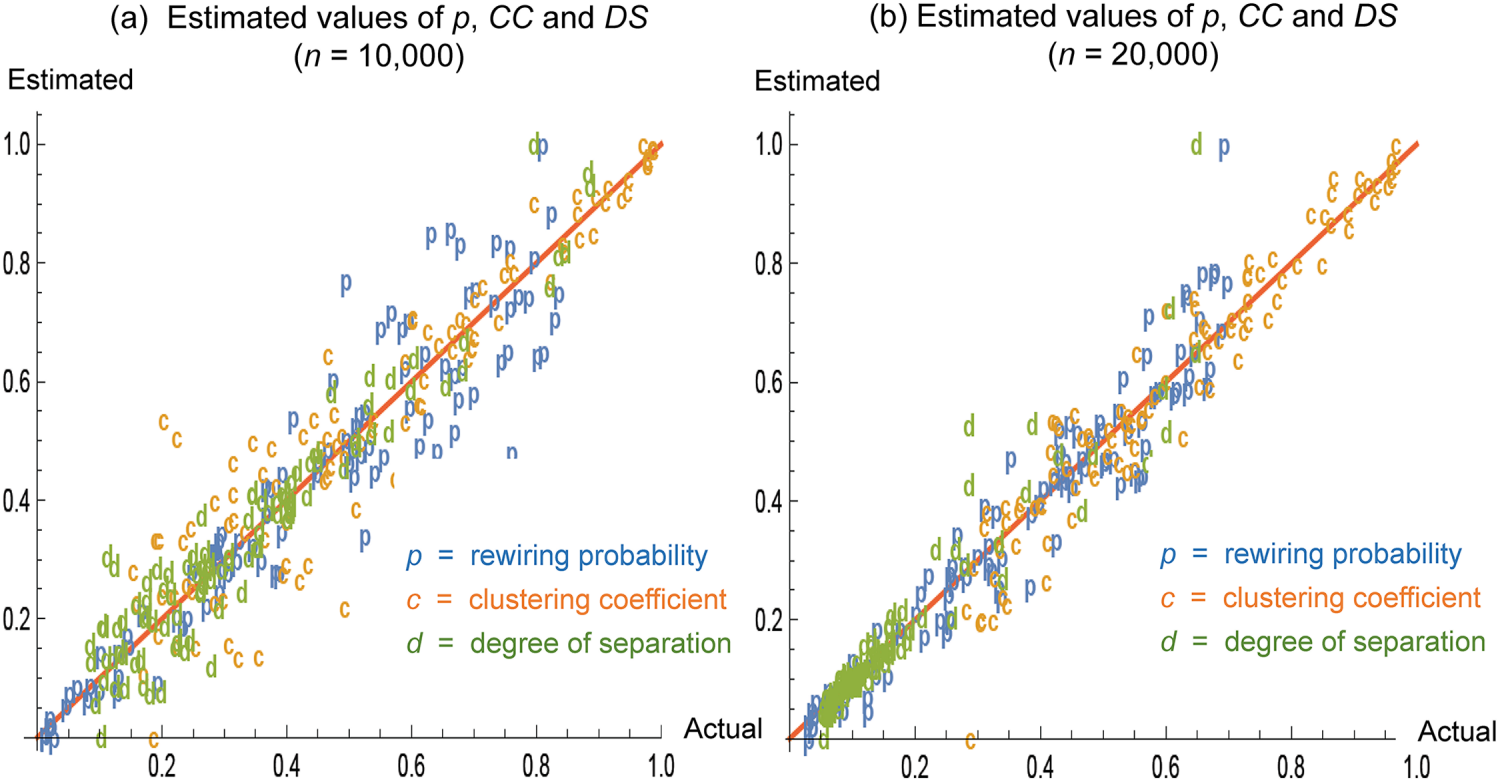
Estimated values of *p*, *CC* and *DS*. For each case of (a) *n* = 10,000 and (b) *n* = 20,000, 100 WS networks were generated with *K* = 80 and a randomly chosen *p* ∈ [0.005, 0.05]. From each network, *s* = 100 nodes were randomly sampled along with their degrees to calculate $\hat{p}$ from Algorithm 1. All estimated values were normalized between 0 and 1.

Fig 4 shows results from application of Algorithm 1 to 200 WS networks (100 networks for *n* = 10,000 and 100 networks for *n* = 20,000) with *K* = 80 and a randomly chosen *p* ∈ [0.005, 0.05]. From each network, *s* = 100 nodes were randomly sampled along with their degrees to calculate $\hat{p}$ from Algorithm 1. The closer the labels are to the diagonal, the more accurate the estimated values match the actual ones. In Fig 4, we normalized all estimated values between 0 and 1 to plot them together. Closer matches between the estimated and actual values are observed for larger networks with *n* = 20,000. The variations around the diagonal seem to be consistent with those in Fig 3, but exacerbated slightly due to the use of $\hat{p}$ as an estimate of *p*.

**Fig 5:**
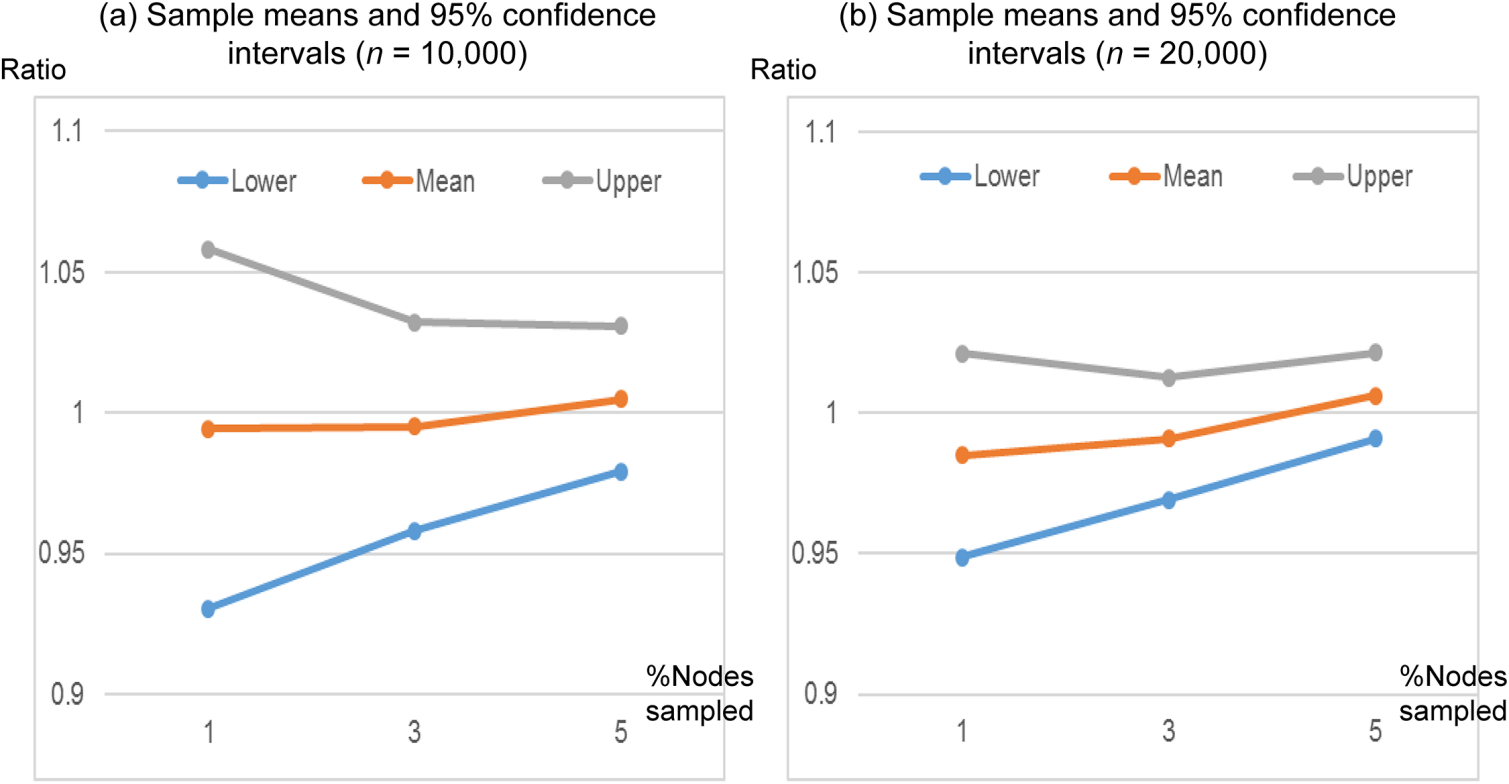
Sample means and 95% confidence intervals of 30 ratios of $\hat{p}/p$. For each combination of *n* = 10,000, 20,000, *K* = 80 and *s*/*n* = 1%, 3%, 5%, 30 WS networks were generated with a randomly chosen *p* ∈ [0.05, 0.2]. For each network, the value of $\hat{p}$ was calculated from Algorithm 1.

In Fig 5, we measure the accuracy of $\hat{p}$ calculated by Algorithm 1. For each combination of *n* = 10,000, 20,000, *K* = 80 and *s*/*n* (percentage of nodes sampled) = 1%, 3%, 5%, we generated 30 WS networks with a randomly chosen *p* ∈ [0.05, 0.2] and calculated $\hat{p}$ from Algorithm 1. Then, for each combination, we calculated the sample mean of 30 ratios ($\hat{p}/p$). The ratio of 1 represents an exact match between $\hat{p}$ and *p*. As sample sizes increase from 1% to 5% of nodes sampled, mean ratios of $\hat{p}/p$ approach to 1. Also, as in Fig 4, higher accuracy is observed for larger networks with *n* = 20,000. For each percentage of nodes sampled (*s*/*n* = 1%, 3%, 5%), the confidence interval for *n* = 20,000 is narrower than that for *n* = 10,000 while both of the confidence intervals overlap with $\hat{p}/p=1$.

Table 1 summarizes the sample means and 95% confidence intervals (in a format of sample mean ± margin of error) in Fig 5. A margin of error is calculated as $t_{0.025}\left(\sigma_{r}/\sqrt{30}\right)$, where *σ*_*r*_ is the sample standard deviation of 30 ratios. Again, For each percentage of nodes sampled (*s*/*n* = 1%, 3%, 5%), the margins of error for *n* = 20,000 are smaller resulting in narrower confidence intervals. Thus, our method adds more accuracy for larger networks (e.g., large-scale social networks).

**Table 1:** Sample means and 95% confidence intervals of 30 ratios of $\hat{p}/p$.

*n*	*s*/*n* = 1%	*s*/*n* = 3%	*s*/*n* = 5%
10,000	0.9943 ± 0.0638	0.9952 ± 0.0371	1.0050 ± 0.0258
20,000	0.9850 ± 0.0362	0.9908 ± 0.0218	1.0061 ± 0.0152

Table notes 95% confidence intervals are shown in a format of sample mean ± margin of error. For each combination of *n* = 10,000, 20,000, *K* = 80 and *s*/*n* = 1%, 3%, 5%, 30 WS networks were generated with a randomly chosen *p* ∈ [0.05, 0.2]. For each network, the value of $\hat{p}$ was calculated from Algorithm 1.

## Conclusion

We have presented a method to construct a Watts-Strogatz network using a sample from a small-world network with symmetric degree distribution. Our method yields an estimated degree distribution which fits closely with that of a WS network and allows to characterize the population with accurate estimates of network metrics such as clustering coefficient and degree of separation. This is particularly useful when sufficient information on the population is not available due to limited resources and time. As observed, our method is more accurate for larger networks.

An obvious limitation of our method is the symmetry of degree distribution and we admit that many real networks have skewed degree distributions. Applications of our method are also limited to networks revealing strong small-world properties which can be well represented by a WS model, since our method is formulated based on the WS rewiring procedure. For a real network either with non-symmetric degree distribution or with weak small-world properties, we still hope that our method serves as a building block for potential revisions or extensions.

Replicating a large network from a sample allows further experiments on a generated network for more insights on its structure and dynamics. This would be feasible if some fundamental properties (e.g., small-world properties) of the network are identified and formulated in an analytical model (e.g., the WS model). Then, key parameters of the model can be estimated and a full-scale network can be constructed.
